# P078: Epidemiology of extended-spectrum beta-lactamase-producing enterobacteriaceae (ESBL-E) during an epidemic, with screening of patients and healthcare workers

**DOI:** 10.1186/2047-2994-2-S1-P78

**Published:** 2013-06-20

**Authors:** A Agostinho, G Jourdan, G Renzi, C Bonfillon, P Hoffmeyer, S Harbarth, I Uçkay

**Affiliations:** 1 Infection Control Program, Geneva University Hospitals, Geneva, Switzerland; 2 Orthopaedic Surgery, Geneva University Hospitals, Geneva, Switzerland; 3 Central Bacteriology Laboratory, Geneva University Hospitals, Geneva, Switzerland; 4 Occupational Medicine Service, Geneva University Hospitals, Geneva, Switzerland

## Objectives

To determine the nosocomial acquisition rate of ESBL-E among patients and healthcare workers (HCWs) during an epidemic (March 2009 to Nov 2010) in an orthopaedics ward at HUG.

## Methods

Universal screening made by anal swab of all patients on admission and every 2 weeks if screening remained negative. 49 samples were collected from 41 HCW and 60 environmental samples were analysed. Molecular typing was performed on all ESBL-E isolates. If there was more than 97.5% similarity, strains were considered identical.

## Results

Between March 2009 and November 2010, 1’531 admissions occurred to the orthopaedic ward (12’401 patient-days; length of stay of 27 days). Among 565 anal swabs, ESBL-E were detected in 204 samples from 45 patients.

The ESBL-E found were *E. coli* (n=39), *Klebsiella pneumoniae* (n=10), *Enterobacter spp* (n=8), *Citrobacter spp* (n=2), *Morganella morganii* (n=2), and *Proteus vulgaris* (n=1). Two different ESBL-E strains were detected in 6 patients, and 3 others carried three distinct isolates. The ESBL-E transmitted were *E. coli* (14 patients), *K. pneumoniae* (3 patients) and both in 2 patients.

Identical ESBL-E species with epidemiological links were found in 25 cases. Only 9 of these were attributable to the unit. Most positive patients (96% [43/45]) were colonized asymptomatically with ESBL-E.

Among HCWs, 6 samples (12%) were positive. Transmission was only observed between patients, not HCWs.

None of the environmental samples revealed presence of ESBL-E.

## Conclusion

Transmission of ESBL-E strains was only observed between patients. No transmission between HCWs and patients occurred. HCW screening and environmental sampling is not useful during ESBL-E carriage outbreaks.

The main ESBL-E transmitted was *E. coli*.

ESBL-E transmission can occur in units with extended length of stay, questioning the new Swiss policy of abandoning contact precautions for *E. coli*-ESBL carriers.

## Disclosure of interest

None declared

